# Recurrence of Pyoderma Gangrenosum Potentially Triggered by COVID-19 Vaccination

**DOI:** 10.7759/cureus.22625

**Published:** 2022-02-26

**Authors:** Abigale L Clark, Blake Williams

**Affiliations:** 1 College of Osteopathic Medicine, Kansas City University of Medicine and Biosciences, Kansas City, USA; 2 Dermatology, Premier Dermatology, Bentonville, USA

**Keywords:** inflammatory mediators, recurrence covid 19, nonhealing ulcer, pyoderma gangenosum, covid-19 vaccine

## Abstract

Pyoderma gangrenosum (PG) is a rare inflammatory skin disease of unknown origin. As with other vaccines, COVID-19 vaccines have been associated with many cutaneous reactions. Although COVID-19 vaccination is crucial, it is important for dermatologists and other physicians to be aware of the possible cutaneous reactions that can occur following COVID-19 vaccination. In this report, we describe a 73-year-old woman with a personal history of PG who experienced a recurrence after receiving her second dose of the tozinameran vaccine. Although extremely rare, flares of other inflammatory dermatoses, including lichen planus, have been reported following COVID-19 vaccination. Here we discuss the overlap in pathogenesis of PG and COVID-19, proposing possible mechanisms behind this rare phenomenon.

## Introduction

Although COVID-19 vaccines are essential tools to combat the pandemic, it is imperative for dermatologists to be aware of the dermatologic reactions that may occur following vaccination. Many dermatologic reactions following COVID-19 vaccination have been reported, but the most common reactions include delayed large local reactions, local injection site reactions, morbilliform reactions, urticaria, and pernio-like lesions. Less common cutaneous reactions include erythema multiforme, lichen planus, pityriasis rosea-like reactions, and purpuric and petechial rashes [1]. It is important to recognize that many of the dermatologic reactions to the vaccine, such as erythema multiforme and pityriasis rosea, mimic the recognized manifestations of natural COVID-19 infection [1]. Notably, the most prevalent patient population reporting cutaneous reactions following vaccination is female patients below the age of 65. As women naturally have a stronger immune response to vaccines and foreign antigens, it is not surprising that women are the most affected patient population [1]. Adding to the body of knowledge regarding dermatologic reactions to COVID-19 vaccination, we herein describe a flare of previously well-controlled pyoderma gangrenosum (PG) occurring following COVID-19 vaccination in an elderly female. It is relevant to mention that there has been one case of previously well-controlled lichen planus, another inflammatory dermatosis, that flared within 48 hours of receiving the COVID-19 mRNA vaccine [2]. Of note, there have been two cases of pyoderma gangrenosum reported after natural infection with COVID-19 in previously healthy patients [3,4]. For reference, the estimated prevalence of pyoderma gangrenosum is 0.0058%, or 5.8 cases per 100,000 adults in the United States [5].

## Case presentation

**Figure 1:**
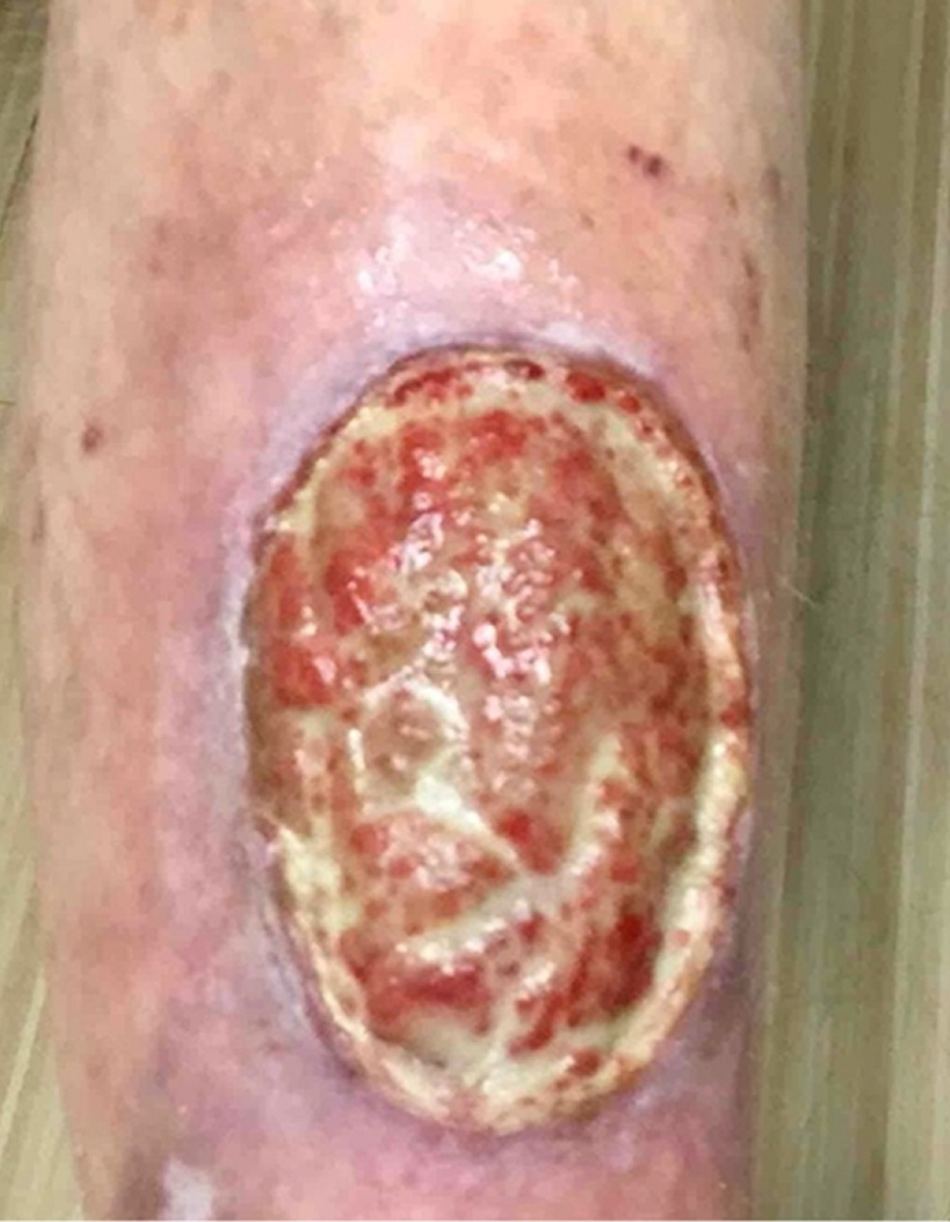
Pyoderma gangrenosum: a large ulcer with surrounding erythema and an irregular, raised purple border. Our patient presented with this ulcer three years prior to the COVID-19 vaccination. This ulcer was successfully treated and closed.

**Figure 2:**
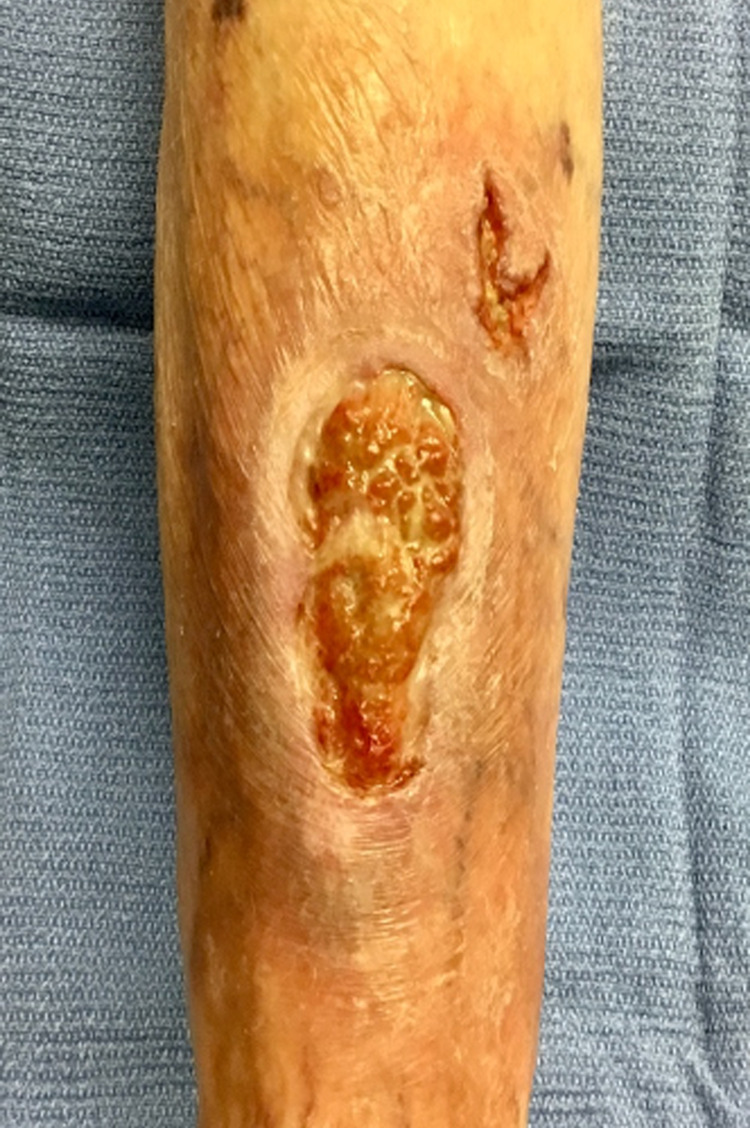
Pyoderma gangrenosum: a large ulcer with surrounding erythema and an irregular, raised border with a purple hue. Our patient presented with this ulcer, in the same location as her previously healed ulcer, shortly after receiving the COVID-19 mRNA vaccine.

A 73-year-old woman with a personal history of pyoderma gangrenosum (Figure 1), who had been successfully treated and closed with a combination of infliximab, cyclosporine, and dapsone three years prior, presented to our dermatology clinic. Upon presentation, she reported that she received her second dose of the tozinameran COVID-19 vaccine one month prior and noticed pain in her pyoderma gangrenosum scar site within a few hours of receiving the vaccine. The patient reported that her left lower leg, at the exact site of previous PG involvement, continued to cause her pain over the following days. Within two weeks after receiving the vaccine, the patient noticed a small ulcer at the scar site where her PG had originally developed three years prior. She reported that the size of her wound continued to increase, as well as her pain level. When asked about possible alternative triggers, the patient denied any changes in medication or increased stress in the weeks prior, as well as any history of leg trauma preceding the development of the ulcer. In addition, the patient was not taking any immunosuppressant drugs when she received her vaccination. A physical exam revealed a large ulcer with surrounding erythema and an irregular, raised purple border consistent with pyoderma gangrenosum on the left pretibial region (Figure 2). Although the patient previously responded well to infliximab injections, retreatment with infliximab resulted in an injection reaction. Her disease remains refractory to oral prednisone, cyclosporine, and biweekly adalimumab injections.

## Discussion

Our knowledge about dermatologic manifestations of the COVID-19 virus and vaccine continues to evolve. Awareness of the diverse array of cutaneous reactions to the COVID-19 vaccine, as well as the dermatologic manifestations of COVID-19 infection, is a crucial step in attempting to understand the overall pathophysiology of this novel virus. Although the pathogenesis underlying cutaneous reactions to the COVID-19 vaccine and virus continues to be investigated, the case presented above suggests that the COVID-19 spike protein may serve as a possible immune trigger for pyoderma gangrenosum. This suggestion is further supported by two existing reports of pyoderma gangrenosum that occurred after natural infection with COVID-19 [3,4]. Although the cause of pyoderma gangrenosum is not completely understood, it is thought to involve a mix of inflammatory dysregulation, neutrophil dysfunction, and genetic mutations. Pyoderma gangrenosum is also associated with an increase in a variety of proinflammatory cytokines, including IL-12, IL-23, tumor necrosis factor (TNF)-alpha, and IL-6 [6]. Interestingly, increased secretion of pro-inflammatory cytokines including IL-1, IL-2R, IL-6, and TNF-alpha also play a key role in the pathogenesis of COVID-19 [7], and increased levels of IL-2R, TNF-alpha, and interferon (IFN)-gamma have been reported in response to the vaccine [2]. Additionally, dysregulation of the Janus Kinase (JAK)/signal transducer and activator of transcription (STAT) signaling pathway is known to play a major role in the inflammatory response induced by COVID-19 [7]. Similarly, several cases of pyoderma gangrenosum have been associated with mutations in Janus Kinase 2, which is an important component of the JAK/STAT pathway [6]. As illustrated by the similarities in both the inflammatory cytokines and signaling pathways involved in the two conditions, it is reasonable to postulate that the introduction of COVID-19 viral antigen, either by vaccine or natural infection, may serve as a potential immune trigger for pyoderma gangrenosum. Due to the significant overlap in the pathogenesis of pyoderma gangrenosum and COVID-19, it is important to consider that certain patients may be at risk of developing pyoderma gangrenosum after exposure to the COVID-19 viral antigen, either through natural infection or vaccination. This risk is most important to consider in patients with a history of pyoderma gangrenosum or other inflammatory conditions.

## Conclusions

We report a case of pyoderma gangrenosum recurrence potentially triggered by COVID-19 vaccination. Although it is difficult to prove causation in any cutaneous vaccine or drug reaction, other potential triggers for recurrence, including stress, trauma, or medication changes, were ruled out. In addition, the patient was not taking any immunosuppressant drugs at the time of this event. The purpose of this report is to contribute to the existing body of knowledge concerning cutaneous side effects that are possible after the COVID-19 vaccination. Although the benefits of vaccination far outweigh the risks, clinicians should be aware of the potential for recurrence of pyoderma gangrenosum and other inflammatory dermatoses after COVID-19 vaccination. Further investigation of this topic is warranted in order to establish a causative relationship.
